# Supplementary material for: Out-of-pocket prescription medicine expenditure amongst community-dwelling adults: Findings from the Irish longitudinal study on ageing (TILDA) in 2016
Source: Explor Res Clin Soc Pharm. 2025 Jan 20;17:100565. doi: 10.1016/j.rcsop.2025.100565 (PMC11833648; doi:10.1016/j.rcsop.2025.100565)
Supplement: Supplementary file 1 — Supplementary material [file mmc1.docx]

**APPENDICES FOR:**

**Out-of-pocket prescription medicine expenditure amongst community-dwelling adults: findings from The Irish Longitudinal Study on Ageing**

By: [REDACTED]

Contents

[eBox 1. Healthcare Coverage in Ireland 3](#_Toc179990446)

[eBox 2. Medicine questions in TILDA wave 4 4](#_Toc179990447)

[eTable 1. Prescription medicine expenditure outlier management 6](#_Toc179990448)

[eBox 4. Health coverage questions 7](#_Toc179990449)

[eBox 5. Question about health conditions in TILDA 8](#_Toc179990450)

[eBox 6. Conditions included in analysis (partly based on Ryan and colleagues) 10](#_Toc179990451)

[eBox 7. International Classification of Primary Care (ICPC) Classification of Body Systems 12](#_Toc179990452)

[eBox 8. Healthcare Expenditure Questions 13](#_Toc179990453)

[eTable 1. Out-of-pocket prescription medicine expenditure for individual conditions 16](#_Toc179990454)

[eTable 2. Logit and generalised linear models assessing associations with out-of-pocket (OOP) prescription medicine expenditure (chronic conditions included as categorical variable) 17](#_Toc179990455)

[eTable 2. Logit and generalised linear models assessing associations with out-of-pocket (OOP) prescription medicine expenditure (equivalised household income included in model) 18](#_Toc179990456)

[eTable 3. Demographic and entitlement characteristics of sample broken down by alternative measures of out-of-pocket prescription medicine expenditure 19](#_Toc179990457)

[eTable 4. Model assessing associations with cost-related non-adherence (CRNA) with chronic conditions included as categorical variable 21](#_Toc179990458)

[eTable 5. Univariate logistic model assessing associations with cost-related non-adherence (CRNA) 22](#_Toc179990459)

# eBox 1. Healthcare Coverage in Ireland

| At the time of data collection for the present study (2016), those with public healthcare entitlements under the General Medical Services (GMS) scheme paid €2.50 per prescribed item, and this expenditure was capped at €25 per household per month.^22^ The GMS scheme is means tested based on income and household structure, with different income thresholds for different age groups. For example, the GMS scheme income threshold for a single person aged 65 years or under is €184 a week (after taxes and allowable expenses).^23^ GMS eligibility can also be provided on a discretionary basis e.g. for severe illness.^24^ For those who are not eligible for the GMS scheme, individuals pay the full price of medications out-of-pocket, but payments are capped at €144 per household per month (during the study period) with the Drugs Payment Scheme (DPS) covering costs in excess of this.^25^  Another major entitlement provision in Ireland is the Long-Term Illness (LTI) scheme which entitles individuals with any one of sixteen specified health conditions (e.g. diabetes, cerebral palsy) to free prescribed medicines to treat that condition.^26^ The LTI scheme is not means tested.^27^ There is less clarity around the medication subsidies provided by private health insurance^28,29^ though they are likely to be limited as most insurance plans are hospital plans.^30^ Other relevant schemes include the *GP Visit Card* which entitles holders to free access to general practitioners (GPs) but provides no specific cover for medicines,^31^ and the Health Amendment Act which entitles those affected by contaminated blood products provided by the State to a similar range of healthcare services to those eligible for the GMS scheme.^32^ Finally, there is also significant tax relief available for health expenses, such as out-of-pocket payments for medicines.^34^ |
| --- |

# eBox 2. Medicine questions in TILDA wave 4

| **HU035: Not counting health insurance refunds, on average about how much [do/does] [you/he/she] pay out-of-pocket for [your/his/her] prescribed drugs per month?**  IWER: IF RESPONDENT DOES NOT PURCHASE PRESCRIBED DRUGS REGULARLY, ASK FOR TOTAL SPENT IN THE LAST 12 MONTHS IN PRESCRIBED DRUGS AND DIVIDE BY 12.  IWER: IF R CANNOT GIVE EXACT VALUE, ACCEPT APPROXIMATE VALUE  VALUE [Allow Responses up to 2 decimal places]  €0.00 … €5,000.00  -98. DK  -99. RF  NOTE: Include the €2.50 per prescribed item charges for medical card holders.  NOTE: Do not consider expenses for self-medication or drugs not prescribed  NOTE: By ‘out of pocket’ expenses we mean everything that is not paid by the insurance company. If [you/he/she] first [pay/pays] but later get it refunded, this is not out of pocket expenses.  (SHARE)  **MD001a:** **Now I would like to record all medications that [you/Rname] [take/takes] on a regular basis, like every day or every week. This will include prescription and non-prescription medications, over-the-counter medicines, vitamins, and herbal and alternative medicines.**  **Do/Does [you/Rname] take any medication on a regular basis?**  1. Yes **GO TO MD001**  5. No  98. DK  99. RF  **IF INTSTATUSW4=1,2 OR 3 & MD001A = 5 GO TO MD008**  **IF INTSTATUSW4= 4 OR 5 & MD001A = 5 GO TO NEXT SECTION**  **IWER: ASK RESPONDENT IF YOU COULD SEE THE MEDICATIONS HE/SHE TAKES SO YOU CAN COPY DOWN THE CORRECT SPELLING OF EACH TABLET.**  **IWER: PROMPT: DO I HAVE ALL OF [YOUR/RName’s] MEDICATIONS HERE?**  **DISPLAY NOTE: INPUT THE BRAND NAME WHEN AVAILABLE, RATHER THAN THE GENERIC NAME FOR ANY MEDICATIONS**  **MD001: TYPE THE FIRST FOUR LETTERS OF THE MEDICATION. YOU WILL BE GIVEN A LIST OF POSSIBILITIES CHOOSE ONE. Each medication is recorded in a separate variable**  **(MD001_01 – MD001_20; MD001_ATC_01 - MD001_ATC_20; MD001_NONPROP_01 - MD001_NONPROP_20)**  95. Other (specify) GO TO MD001oth  98. DK  99. RF  **MD001oth: Other (specify)**  **IF THE MEDICATION DOES NOT APPEAR ON THE LIST CAREFULLY TYPE THE FULL MEDICATION NAME. MAKE SURE YOU TYPE THE NAME OF THE BRAND USED AND NOT THE CHEMICAL NAME.**  **(MD001OTH_01-MD001OTH_20)**  Text: up to 60 characters GO TO MD005  **MD005: Was this medication prescribed by a doctor or did you get it over the counter? (MD005_01-MD005_20)**  1. Prescribed by a doctor  2. Over the counter  98. DK  99. RF  **(Note to Scripters -** For medications that are available over the counter as well as on prescription, we are interested in how the respondent got them.)  **RETURN AND REPEAT MD001 FOR UP TO 20 MEDICATIONS PER PERSON**  **IF instatusW4= 4 OR 5 GO TO NEXT SECTION**  **MD006: In the last 12 months have you ever forgotten to take any of the medicines you are supposed to regularly take?**  1. Yes GO TO MD007  5. No GO TO MD008  98. DK GO TO MD008  99. RF GO TO MD008  **MD007: How often have you forgotten to take your medication in the last 12 months?**  1. Rarely  2. Sometimes  3. Often  98. DK  99. RF  **MD008: In the last 12 months, have you ever received a prescription from your GP that you didn’t fill with the pharmacy because you thought that the medication was too expensive?**  1. Yes  5. No  98. DK  99. RF |
| --- |

# eTable 1. Prescription medicine expenditure outlier management


| Participant Response | Recoded to | N | Reason for recoding |
| --- | --- | --- | --- |
| €600 | €50 | 1 | Not a medical card holder and therefore, likely to be annual expenditure rather than monthly expenditure, therefore divided by 12 |
| €720 | €60 | 1 | Not a medical card holder and therefore, likely to be annual expenditure rather than monthly expenditure, therefore divided by 12 |
| €1032 | €86 | 1 | Not a medical card holder and therefore, likely to be annual expenditure rather than monthly expenditure, therefore divided by 12 |
| €1728 | €144 | 1 | Not a medical card holder and therefore, likely to be annual expenditure rather than monthly expenditure, therefore divided by 12 |
| >€288 but no logical explanation for being >€288 | Missing | 13 | No logical explanation for being >€288 |
| €1,250 | €12.50 | 2 | Medical card holder (€2.50 per medicine) and prescribed five regular medicines, therefore likely a mistaken decimal place |
| €750 | €7.50 | 3 | Medical card holder (€2.50 per medicine) and prescribed three regular medicines, therefore likely a mistaken decimal place |

# eBox 3. Health coverage questions

| **HU001: [Is/Are] [you/he/she] covered by:**  IWER: CODE THE ONE THAT APPLIES  1. Full Medical Card or equivalent  2. GP Visit Card  96. Neither of these  98. DK  99. RF  **Note:** This question is asked even of those covered by private medical insurance. Most over 70s are entitled to medical cards.  (EU-SILC)  **HU070: [Is/Are] [you/he/she] covered by:**   1. The long term illness scheme 2. A Health Act Amendment Card   96. Neither of these  98 DK  99 RF  **HU002: [Do/Does] [you/he/she] have private medical insurance cover (VHI etc.) in [your/his/her] own name or through another family member?**  1. Yes, in own name GO TO HU003  2. Yes, as the spouse of a subscriber GO TO HU003  3. Yes, as the relative of a subscriber GO TO HU003  5. No GO TO HU049  98. DK GO TO HU049  99. RF GO TO HU049  (HEALTH INSURANCE AUTHORITY 2005 SURVEY) |
| --- |

# eBox 4. Question about health conditions in TILDA

| (A) Since your last interview, has a doctor ever told you/ that you have any of the [other] conditions on this card?  (B) Has a doctor ever told [you/Rname] that [you/he/she] [have/has] any of the conditions on this card?  **IWER: PROBE - 'WHAT OTHERS?' CODE ALL THAT APPLY.**  1. Chronic lung disease such as chronic bronchitis  or emphysema **GO TO PH302 [ph301_01]**  2. Asthma **[ph301_02]**  3. Arthritis (including osteoarthritis, or rheumatism) **GO TO PH304 [ph301_03]**  4. Osteoporosis, sometimes called thin or brittle bones **[ph301_04]**  5. Cancer or a malignant tumour **GO TO PH309 [ph301_05]**  (including leukaemia or lymphoma but excluding minor skin cancers)  6. Parkinson's disease **GO TO PH314 [ph301_06]**  7. Any emotional, nervous or psychiatric problems,  such as depression or anxiety **GO TO PH315 [ph301_07]**  9. Alzheimer's disease **GO TO PH318 [ph301_09]**  10. Dementia, organic brain syndrome, senility **GO** **TO PH319** **[ph301_10]**  11. Serious memory impairment **GO TO PH319a [ph301_11]**  12. Stomach ulcers  **[ph301_12]**  13. Varicose Ulcers (an ulcer due to varicose veins)  **[ph301_13]**  14. Cirrhosis, or serious liver damage  **[ph301_14]**  15. Thyroid Problems **GO TO PH325 [ph301_15]**  16. Alcohol abuse  **GO TO PH320a [ph301_16]**  17. Substance abuse **GO TO PH320b [ph301_17]**  18. Chronic kidney disease **GO TO PH327 [ph301_18]**  19. Severe Anaemia  **[ph301_19]**  20. Epilepsy GO TO PH329 **[ph301_20]**  95. Other (please specify)  **[ph301_95] [ph301oth]**  96. None of these **GO TO PH328 [ph301_96]**  98. DK **GO TO PH328 [ph301_98]**  99. RF **GO TO PH328 [ph301_99]**  (ELSA/ similar question HRS/NSHAP)  **IWER: SHOW CARD PH2 [PAGE #]**  **IF (intstatusW4 = 2, 4, 5), USE WORDING ‘B’. ALL OTHERS, USE WORDING ‘A’.**  PH201: Please look at card PH2.  (A) Since your last interview, has a doctor ever told you that you have any of the [other] conditions on this card?  (B) Has a doctor ever told [you/Rname] that [you/he/she] [have/has] any of the conditions on this card?  INTERVIEWER: PROBE - 'WHAT OTHERS?' CODE ALL THAT APPLY.  1. High blood pressure or hypertension  **[ph201_01]**  2. Angina  **[ph201_02]**  3. A heart attack  (including myocardial infarction or coronary thrombosis) **[ph201_03]**  4. Congestive heart failure  **[ph201_04]**  5. Diabetes or high blood sugar  **[ph201_05]**  6. A stroke (cerebral vascular disease)  **[ph201_06]**  7. Ministroke or TIA  **[ph201_07]**  8. High cholesterol  **[ph201_08]**  9. A heart murmur  **[ph201_09]**  11. Atrial Fibrillation  **[ph201_11]**  12. An abnormal heart rhythm (not atrial fibrillation)  **[ph201_12]**  95. Any other heart trouble (please specify)  **[ph201_95][ph201oth]**  96. None of these  **[ph201_96]**  98. DK  **[ph201_98]**  99. RF  **[ph201_99]**  (ELSA/ similar questions in HRS/ SHARE)  (A) Since [your] last interview, has a doctor ever told you that you have any of the following [other] eye diseases?  (B) Has a doctor ever told [you/Rname] that [you/he/she] [have/has] any of the following eye diseases? [DISPLAY ALL CONDITIONS]  **IWER: READ OUT. CODE ALL THAT APPLY.**  1. Cataracts **[ph105_01]**  2. Glaucoma **[ph105_02]**  3. Age related macular degeneration **[ph105_03]**  95. Other (please specify) **[ph105_95] [ph105oth]** |
| --- |

# eBox 5. Conditions included in analysis (partly based on Ryan and colleagues)

| 1. Cardiac Condition  - Angina - Heart Attack - Congestive Heart Failure - Heart Murmur - Abnormal heart rhythm - Atrial fibrillation - Any heart trouble  1. Cerebrovascular disease  - Stroke - TIA  1. HTN 2. Diabetes 3. High Cholesterol 4. Chronic respiratory disease  - Chronic lung disease - Asthma  1. Liver disease  - Alcohol abuse - Cirrhosis - Liver disease  1. Eye disease  - Cataracts - Glaucoma - Age related macular degeneration - Other eye disease  1. Cognitive Impairment  - Alzheimer’s   - Dementia   - Serious cognitive impairment  1. Arthritis 2. Osteoporosis 3. Cancer 4. Parkinson’s disease 5. Emotional /psychological condition including anxiety and depression 6. Substance abuse 7. Stomach Ulcers 8. Varicose veins including varicose ulcers 9. Epilepsy 10. Thyroid Problems 11. Chronic kidney disease 12. Anaemia |
| --- |

# eBox 6. International Classification of Primary Care (ICPC) Classification of Body Systems

| 1. Cardiovascular 2. Respiratory 3. Digestive 4. Eyesystem 5. Psychological 6. Metabolic 7. Musculoskeletal 8. Neurological 9. Urological |
| --- |

# eBox 7. Healthcare Expenditure Questions

| **HU005: In the last 12 months, about how often did [you/he/she] visit [your/his/her] GP?**  IWER: IF RESPONDENT HAS NOT VISITED GP IN THE LAST 12 MONTHS CODE **0**  0…200  -98. DK GO TO HU007  -99. RF GO TO HU007  IF (HU005=0, -98,-99) GO TO HU007  IF (HU005>0 AND HU001=1, 2) GO TO HU007  IF (HU005>0 AND HU070= 2) GO TO HU007  IF (HU005>0 AND HU001≠1, 2) GO TO HU006  IF (HU005>0 AND HU070 ≠ 2) GO TO HU006  (SHARE)  **HU006: How much did [you/he/she] pay for [your/his/her] last visit to the GP, after any health insurance reimbursement?’**  IWER: IF R CANNOT GIVE EXACT VALUE, ACCEPT APPROXIMATE VALUE  €0.00 … €10,000  -98. DK  -99. RF  **HU038: In total, how much did [you/he/she] pay for all of [your/his/her] A&E visit(s) in the last 12 months, after any health insurance reimbursement?’**  IWER: IF R CANNOT GIVE EXACT VALUE, ACCEPT APPROXIMATE VALUE  €0 … €10,000  -98. DK  -99. RF  **HU075: In total, how much did [you/he/she] pay for [your/his/her] outpatient/day patient visits in the last 12 months, after any health insurance reimbursement?** (May be zero)  IWER: IF R CANNOT GIVE EXACT VALUE, ACCEPT APPROXIMATE VALUE  €0 … €50,000  -98. DK  -99. RF  If HU062=1  **HU039: In total, how much did [you/he/she] pay for [your/his/her] visit(s) to consultant(s) in the last 12 months, after any health insurance reimbursement?** (May be zero)  IWER: IF R CANNOT GIVE EXACT VALUE, ACCEPT APPROXIMATE VALUE  €0 … €20,000  -98. DK  -99. RF  **HU040: In total, how much did [you/he/she] pay for [your/his/her] overnight hospital stays(s) in the last 12 months, after any health insurance reimbursement?** (May be zero)  IWER: IF R CANNOT GIVE EXACT VALUE, ACCEPT APPROXIMATE VALUE  €0 … €50,000  -98. DK  -99. RF  **HOME CARE**  **IF HH002=1 GO TO HU076**  **IF HH002=2, 3 GO TO HU083**  **HU015_a4: Not counting costs paid by the HSE/health board, about how much did [you/Rname] (and [your/his/her] [husband/wife/partner]) pay for this home help in the last month?** (May be zero)  €0 … €10,000  -98. DK  -99. RF  GO TO HU015  **HU015_b4: Not counting costs paid by the HSE/health board, about how much did [you/Rname] (and [your/his/her] [husband/wife/partner]) pay this personal care attendant in the last month?** (May be zero)  €0 … €10,000  -98. DK  -99. RF  GO TO HU015  **HU015_d4: Not counting costs paid by the HSE/health board, about how much did [you/Rname] (and [your/his/her] [husband/wife/partner]) pay for this Home Care Package in the last month?** (May be zero)  €0 … €10,000  -98. DK  -99. RF  GO TO HU015  **HU036: Not counting any refunds from [your/his/her] health insurance, about how much did [you /he/she] pay (out-of-pocket) for any other health expenses [you/he/she] had in the last 12 months?**  €0 … and €20,000  -98. DK  -99. RF  **Note:** By other health expenses we mean non-prescription drugs, private physiotherapy, preventive rehabilitative services such as occupational therapy etc.  By ‘out of pocket’ expenses we mean everything that is not paid by the insurance company. If [you/he/she] first pay/pays] but later [get/gets] it refunded, this is not out of pocket expenses. Prescription drugs should be included in HU035 and not here.  (SHARE) |
| --- |

# eTable 2. Out-of-pocket prescription medicine expenditure for individual conditions

| **Condition** | **% (N)** | **Mean OOP Prescription Medicine Expenditure (SD)** | **Median OOP Prescription Medicine Expenditure (IQR)** |
| --- | --- | --- | --- |
| Hypertension | 37.6% (2,131) | €399 (492) | €240 (120-420) |
| High Cholesterol | 36.8% (2,087) | €390 (502) | €204 (60-456) |
| Arthritis | 35.7% (2,026) | €371 (503) | €180 (60-360) |
| Eye Disease | 17.1% (970) | €378 (513) | €192 (90-324) |
| Osteoporosis | 15.9% (903) | €404 (517) | €240 (90-444) |
| Cardiac Condition | 14.2% (805) | €462 (557) | €240 (120-480) |
| Chronic Respiratory Disease | 11.2% (637) | €526 (594) | €300 (120-720) |
| Thyroid Problems | 9.2% (524) | €431 (482) | €282 (120-540) |
| Diabetes | 9.0% (512) | €292 (448) | €150 (24-300) |
| Emotional/Psychological Condition including Anxiety and Depression | 7.5% (423) | €399 (478) | €240 (120-360) |
| Gastrointestinal Conditions including Stomach Ulcers | 2.9% (163) | €382 (473) | €240 (120-336) |
| Cancer | 2.7% (152) | €447 (568) | €198 (90-540) |
| Varicose Veins including Varicose Ulcers | 1.7% (96) | €302 (399) | €180 (87-300) |
| Cerebrovascular Disease | 1.2% (69) | €516 (717) | €204 (90-480) |
| Chronic Kidney Disease | 0.8% (44) | €452 (519) | €144 (0-312) |
| Liver Disease | 0.7% (42) | €354 (459) | €240 (72-360) |
| Parkinson’s Disease | 0.6% (36) | €417 (640) | €222 (15-360) |
| Cognitive Impairment | N.A. (<30) | N.A. | N.A. |
| Epilepsy | N.A. (<30) | N.A. | N.A. |
| Substance Abuse | N.A. (<30) | N.A. | N.A. |
| Anaemia | N.A. (<30) | N.A. | N.A. |

# eTable 3. Logit and generalised linear models assessing associations with out-of-pocket (OOP) prescription medicine expenditure (chronic conditions included as categorical variable)

|  | Any OOP expenditure (N=5,513) |  | Value of OOP expenditure^a^ (N=4,050) |  |
| --- | --- | --- | --- | --- |
|  | Odds ratio (95%CI) | p value | Rate ratio (95%CI) | p value |
| **Healthcare entitlements (Ref: GMS scheme)** |  |  |  |  |
| GP visit card-holder | 0.79 (0.55-1.17) | 0.232 | 4.68 (4.23-5.19) | <0.001 |
| Neither GMS scheme nor GP visit card | 0.62 (0.47-0.83) | 0.001 | 4.74 (4.36-5.16) | <0.001 |
| **LTI scheme**^b^ | 0.67 (0.46-0.97) | 0.035 | 0.99 (0.89-1.11) | 0.919 |
| **Private Health Insurance**^b^ | 1.10 (0.85-1.43) | 0.458 | 1.07 (0.99-1.15) | 0.081 |
| **Age (years)** | 1.00 (0.99-1.02) | 0.804 | 1.01 (1.00-1.01) | 0.006 |
| **Female sex**^b^ | 0.75 (0.61-0.93) | 0.008 | 0.92 (0.86-0.97) | 0.006 |
| **Education (Ref: primary/none)** |  |  |  |  |
| Secondary | 0.73 (0.54-0.99) | 0.043 | 1.00 (0.93-1.09) | 0.929 |
| Third/higher | 0.79 (0.57-1.10) | 0.165 | 1.02 (0.94-1.11) | 0.604 |
| **Number of conditions (ref: 0 conditions)** |  |  |  |  |
| 1 | 2.15 (1.62-2.84) | <0.001 | 1.03 (0.91-1.16) | 0.666 |
| 2 | 3.01 (2.20-4.13) | <0.001 | 1.12 (0.99-1.26) | 0.084 |
| 3+ | 3.46 (2.19-5.47) | <0.001 | 1.26 (1.09-1.46) | 0.002 |
| **Complex multimorbidity**^b^ | 1.18 (0.76-1.82) | 0.460 | 1.02 (0.91-1.13) | 0.764 |
| **Number of Prescription Medicines (Ref: 2-3 medicines)** |  |  |  |  |
| 0 medicines | 0.01 (0.01-0.01) | <0.001 | 0.94 (0.81-1.09) | 0.414 |
| 1 medicine | 0.34 (0.25-0.47) | <0.001 | 0.65 (0.60-0.71) | <0.001 |
| 4-5 medicines | 1.18 (0.75-1.87) | 0.475 | 1.44 (1.33-1.56) | <0.001 |
| ≥6 medicines | 0.75 (0.48-1.19) | 0.226 | 1.93 (1.76-2.11) | <0.001 |
| **Urban**^b^ | 1.01 (0.82-1.24) | 0.929 | 1.11 (1.04-1.17) | 0.001 |
| **Partnered**^b^ | 1.06 (0.83-1.34) | 0.653 | 1.03 (0.97-1.11) | 0.328 |
| Intercept | 17.07 (4.74-61.41) | <0.001 | 80.90 (56.87-115.08) | <0.001 |

^a^Model b includes only those with any out-of-pocket prescription medicine expenditure

Note: The variables are mutually adjusted

^b^Reference groups where not indicated are no LTI scheme eligibility, no private health insurance, male sex, no complex multimorbidity, not urban, and not partnered.

Abbreviations: CI, confidence interval; GMS, General Medical Services; GP, general practitioner; LTI, Long Term Illness

# eTable 4. Logit and generalised linear models assessing associations with out-of-pocket (OOP) prescription medicine expenditure (equivalised household income included in model)

|  | Any OOP expenditure (N=4,836) |  | Value of OOP expenditure^a^ (N=3,567) |  |
| --- | --- | --- | --- | --- |
|  | Odds ratio (95%CI) | p value | Rate ratio (95%CI) | p value |
| **Healthcare entitlements (Ref: GMS scheme)** |  |  |  |  |
| GP visit card-holder | 0.75 (0.49-1.15) | 0.188 | 4.99 (4.48-5.56) | <0.001 |
| Neither GMS scheme nor GP visit card | 0.62 (0.45-0.85) | 0.003 | 4.92 (4.49-5.39) | <0.001 |
| **LTI scheme**^b^ | 0.69 (0.46-1.03) | 0.072 | 1.01 (0.90-1.14) | 0.804 |
| **Private health insurance**^b^ | 1.10 (0.84-1.44) | 0.495 | 1.05 (0.97-1.13) | 0.246 |
| **Age (years)** | 1.00 (0.99-1.02) | 0.672 | 1.01 (1.00-1.01) | 0.005 |
| **Female sex**^b^ | 0.70 (0.56-0.88) | 0.002 | 0.92 (0.86-0.98) | 0.007 |
| **Equivalised household income (Ref: Quintile 1, €0-€6,000)** |  |  |  |  |
| €6,006-€10,000 | 1.35 (0.93-1.95) | 0.119 | 0.98 (0.89-1.08) | 0.735 |
| €10,029-€15,133 | 1.07 (0.74-1.55) | 0.707 | 1.05 (0.95-1.15) | 0.351 |
| €15,142-€24,000 | 0.97 (0.67-1.41) | 0.890 | 0.97 (0.87-1.08) | 0.586 |
| €24,001-€270,000 | 1.04 (0.70-1.53) | 0.860 | 1.03 (0.92-1.15) | 0.642 |
| **Number of conditions** | 1.46 (1.29-1.65) | <0.001 | 1.07 (1.03-1.10) | <0.001 |
| **Complex multimorbidity**^b^ | 0.82 (0.54-1.24) | 0.347 | 1.00 (0.91-1.11) | 0.932 |
| **Number of Prescription Medicines (Ref: 2-3 medicines)** |  |  |  |  |
| 0 medicines | 0.01 (0.01-0.01) | <0.001 | 0.88 (0.75-1.03) | 0.125 |
| 1 medicine | 0.33 (0.23-0.46) | <0.001 | 0.66 (0.61-0.72) | <0.001 |
| 4-5 medicines | 1.24 (0.75-2.06) | 0.408 | 1.44 (1.32-1.56) | <0.001 |
| ≥6 medicines | 0.69 (0.42-1.12) | 0.132 | 1.92 (1.75-2.11) | <0.001 |
| **Urban**^b^ | 0.98 (0.79-1.23) | 0.888 | 1.10 (1.03-1.17) | 0.003 |
| **Partnered**^b^ | 1.05 (0.82-1.36) | 0.689 | 1.04 (0.97-1.12) | 0.315 |
| Intercept | 17.78 (4.72-66.98) | <0.001 | 77.71 (54.88-110.04) | <0.001 |

^a^Model b includes only those with any out-of-pocket prescription medicine expenditure

Note: The variables are mutually adjusted

^b^Reference groups where not indicated are no LTI scheme eligibility, no private health insurance, male sex, no complex multimorbidity, not urban, and not partnered.

Abbreviations: CI, confidence interval; GMS, General Medical Services; GP, general practitioner; LTI, Long Term Illness

# eTable 5. Demographic and entitlement characteristics of sample broken down by alternative measures of out-of-pocket prescription medicine expenditure

|  | Proportion of out-of-pocket healthcare expenditure spent on prescription medicines Mean (SD) | Proportion of out-of-pocket healthcare expenditure spent on prescription medicines Median (IQR) | Financial burden  Mean (SD) | Financial burden  Median (IQR) |
| --- | --- | --- | --- | --- |
| Age (years) |  |  |  |  |
| <60 | 51.8% (41.4%) | 61.5% (0.0-95.2) | 2.5% (7.6%) | 0.4% (0.0-2.5) |
| 60-69 | 62.4% (38.6%) | 75.1% (28.6—100.0) | 3.2% (8.0%) | 1.1% (0.0-3.3) |
| 70-79 | 79.2% (32.0%) | 100.0% (64.7-100.0) | 3.9% (10.2%) | 1.6% (0.5-3.8) |
| 80-89 | 85.5% (26.9%) | 100.0% (84.2-100.0) | 5.2% (11.3%) | 2.5% (1.0-5.3) |
| 90+ | 91.9% (20.7%) | 100.0% (100.0-100.0) | 6.6% (15.2%) | 2.2% (0.8-4.4) |
| Sex |  |  |  |  |
| Female | 68.4% (37.7%) | 85.2% (41.9-100.0) | 3.8% (9.4%) | 1.4% (0.0-3.8) |
| Male | 68.4% (38.0%) | 87.0% (40.0-100.0) | 3.3% (8.9%) | 1.1% (0.0-3.1) |
| Education |  |  |  |  |
| Primary/none | 84.9% (29.7%) | 100.0% (87.8-100.0) | 4.2% (10.4%) | 1.7% (0.5-3.8) |
| Secondary | 67.3% (38.4%) | 84.4% (37.5-100.0) | 3.5% (9.0%) | 1.2% (0.0-3.4) |
| Third/higher | 56.7% (38.4%) | 70.6% (21.6-99.6) | 3.2% (8.4%) | 1.0% (0.0-3.3) |
| Equivalised household income (quintiles)* |  |  |  |  |
| €0-€6,000 | 83.7% (29.8) | 100.0% (80.0-100.0) | 8.7% (18.1%) | 3.3% (1.1-6.9) |
| €6,006-€10,000 | 76.0% (35.5) | 100.0% (59.0-100.0) | 3.1% (4.6%) | 0.4% (0.4-3.6) |
| €10,029-€15,133 | 70.3% (37.1) | 91.1% (44.1-100.0) | 2.7% (4.0%) | 0.2% (0.0-2.7) |
| €15,142-€24,000 | 60.1% (38.9) | 73.9% (23.9-100.0) | 1.9% (2.8%) | 0.0% (0.0-2.4) |
| €24,001-€270,000 | 52.0% (38.0) | 60.9% (0.00-84.8) | 1.2% (1.7%) | 0.0% (0.0-1.6) |
| Area of residence |  |  |  |  |
| Urban | 67.7% (37.3%) | 82.8% (40.0-100.0) | 3.9% (10.2%) | 1.4% (0.0-3.8) |
| Not Urban | 69.0% (38.6%) | 91.1% (40.0-100.0) | 3.1 (7.7%) | 1.2% (0.0-3.2) |
| Marital Status |  |  |  |  |
| Partnered | 64.7% (38.5%) | 78.9% (33.3-100.0) | 3.0% (7.8%) | 1.1% (0.0-3.1) |
| Not Partnered | 76.5% (34.9%) | 100.0 (58.3-100.0) | 4.7% (11.5%) | 1.8% (0.3-4.5) |
| Private Health Insurance |  |  |  |  |
| Yes | 57.5% (37.8%) | 66.7% (23.1-95.6) | 3.7% (9.5%) | 1.2% (0.0-3.7) |
| No | 86.1% (30.6%) | 100.0% (99.7-100.0) | 3.3% (8.6%) | 1.4% (0.2-3.3) |
| LTI Scheme |  |  |  |  |
| Yes | 71.9% (37.9%) | 100.0% (46.2-100.0) | 4.2% (11.5%) | 1.3% (0.1-3.7) |
| No | 68.1% (37.8%) | 85.4% (40.0-100.0) | 3.5% (8.9%) | 1.3% (0.0-3.5) |
| GMS & GP Entitlements |  |  |  |  |
| GMS Scheme | 87.4% (27.4%) | 100.0% (100.0-100.0) | 3.1% (9.1%) | 1.4% (0.4-3.0) |
| GP Visit Card-Holder | 79.0% (28.7%) | 91.5% (66.7-100.0) | 6.0% (9.7%) | 3.1% (1.0-7.4) |
| Neither | 45.4% (37.0%) | 54.4% (0.0-79.2) | 3.4% (9.0%) | 0.8% (0.0-3.4) |
| Number of Prescription Medicines (quintiles) |  |  |  |  |
| 0 regular medicines | 16.7% (34.8%) | 0.00 (0.00-0.00) | 0.4% (1.6%) | 0.0% (0.0-0.0) |
| 1 regular medicine | 68.9% (33.6%) | 78.3% (44.4-100.0) | 2.2% (6.1%) | 0.8% (0.3-2.1) |
| 2-3 regular medicines | 78.6% (27.7%) | 93.4% (63.2-100.0) | 4.2% (10.4%) | 1.7% (0.8-3.9) |
| 4-5 regular medicines | 83.9% (24.2%) | 100.0 (72.7-100.0) | 5.3% (9.9%) | 2.8% (1.4-5.5) |
| 6+ regular medicines | 87.4% (22.5%) | 100.0% (82.5-100.0) | 7.4% (13.4%) | 4.0% (2.0-7.2) |
| Number of Chronic Conditions |  |  |  |  |
| 0 chronic conditions | 37.5% (44.0%) | 0.0% (0.0-92.3) | 1.2% (5.7%) | 0.0% (0.0-0.5) |
| 1 chronic condition | 62.5% (39.1%) | 75.0 (28.6-100.0) | 2.4% (6.2%) | 0.7% (0.0-2.5) |
| 2 chronic conditions | 72.8% (34.2%) | 88.9% (53.7-100.0) | 4.0% (10.1%) | 1.5% (0.5-3.7) |
| 3+ chronic conditions | 80.2% (29.3%) | 100.0 (65.8-100.0) | 5.2% (11.1%) | 2.5% (1.0-5.3) |
| Complex Multimorbidity |  |  |  |  |
| Yes | 80.6% (28.8%) | 100.0% (23.5-100.0) | 5.4% (11.4%) | 2.6% (1.1-5.5) |
| No | 62.8% (40.1%) | 78.3% (23.5-100.0 | 2.8% (7.9%) | 0.8% (0.0-2.6%) |

# eTable 6. Model assessing associations with cost-related non-adherence (CRNA) with chronic conditions included as categorical variable

|  | Reporting CRNA (N=5,096) |  |
| --- | --- | --- |
|  | Odds ratio (95%CI) | p value |
| **Any out-of-pocket prescription medicine expenditure (Ref: no out-of-pocket prescription medicine expenditure)** | 0.78 (0.33-1.85) | 0.567 |
| **Out-of-pocket prescription medicine expenditure (per €100)** | 0.99 (0.93-1.05) | 0.685 |
| **Any out-of-pocket healthcare (excl. prescription medicines) expenditure** | 1.29 (0.68-2.44) | 0.429 |
| **Out-of-pocket healthcare (excl. prescription medicines) expenditure (per €100)** | 1.01 (0.98-1.04) | 0.622 |
| **Healthcare entitlements (Ref: GMS scheme)** |  |  |
| GP visit card-holder | 1.67 (0.69-4.03) | 0.254 |
| Neither GMS scheme nor GP visit card-holder | 1.62 (0.74-3.56) | 0.230 |
| **LTI scheme**^a^ | 1.26 (0.59-2.71) | 0.550 |
| **Private Health Insurance**^a^ | 0.62 (0.35-1.07) | 0.087 |
| **Age (years)** | 0.99 (0.95-1.02) | 0.352 |
| **Female sex**^a^ | 1.11 (0.70-1.76) | 0.668 |
| **Education (Ref: primary/none)** |  |  |
| Secondary | 1.33 (0.72-2.43) | 0.362 |
| Third/higher | 1.07 (0.55-2.12) | 0.836 |
| **Number of conditions (Ref: 0 conditions)** |  |  |
| 1 | 1.21 (0.48-3.09) | 0.688 |
| 2 | 1.32 (0.51-3.41) | 0.567 |
| 3+ | 1.19 (0.38-3.75) | 0.763 |
| **Complex multimorbidity**^a^ | 1.77 (0.79-3.98) | 0.165 |
| **Number of Prescription Medicines (Ref: 2-3 medicines)** |  |  |
| 0 medicines | 0.53 (0.19-1.51) | 0.236 |
| 1 medicine | 0.95 (0.46-1.95) | 0.883 |
| 4-5 medicines | 1.89 (1.03-3.46) | 0.039 |
| ≥6 medicines | 1.12 (0.52-2.39) | 0.771 |
| **Urban**^a^ | 1.25 (0.80-1.95) | 0.336 |
| **Partnered**^a^ | 0.68 (0.42-1.10) | 0.117 |
| Intercept | 0.03 (0.00-0.44) | 0.011 |

Note: The variables are mutually adjusted.

^a^Reference groups where not indicated are no LTI scheme eligibility, no private health insurance, male sex, no complex multimorbidity, not urban, and not partnered.

Abbreviations: CI, confidence interval; GMS, General Medical Services; GP, general practitioner; LTI, Long Term Illness

# eTable 7. Model assessing associations with cost-related non-adherence (CRNA) with equivalised household income included

|  | Reporting CRNA (N=4,473) |  |
| --- | --- | --- |
|  | Odds ratio (95%CI) | p value |
| **Any out-of-pocket prescription medicine expenditure (Ref: no out-of-pocket prescription medicine expenditure)** | 0.68 (0.28-1.67) | 0.396 |
| **Out-of-pocket prescription medicine expenditure (per €100)** | 0.98 (0.92-1.05) | 0.520 |
| **Any out-of-pocket healthcare (excl. prescription medicines) expenditure** | 1.46 (0.74-2.86) | 0.273 |
| **Out-of-pocket healthcare (excl. prescription medicines) expenditure (per €100)** | 1.01 (0.97-1.05) | 0.726 |
| **Healthcare entitlements (Ref: GMS scheme)** |  |  |
| GP visit card-holder | 2.54 (1.01-6.39) | 0.047 |
| Neither GMS scheme nor GP visit card-holder | 2.24 (0.97-5.19) | 0.059 |
| **LTI scheme**^a^ | 1.39 (0.64-3.04) | 0.405 |
| **Private Health Insurance**^a^ | 0.53 (0.30-0.94) | 0.029 |
| **Age (years)** | 0.99 (0.95-1.02) | 0.387 |
| **Female sex**^a^ | 1.07 (0.65-1.76) | 0.779 |
| **Equivalised household income (Ref: Quintile 1, €0-€6,000)** |  |  |
| €6,006-€10,000 | 1.26 (0.64-2.48) | 0.496 |
| €10,029-€15,133 | 1.07 (0.53-2.13) | 0.856 |
| €15,142-€24,000 | 0.49 (0.20-1.17) | 0.108 |
| €24,001-€270,000 | 0.45 (0.18-1.15) | 0.097 |
| **Number of conditions** | 1.07 (0.85-1.34) | 0.581 |
| **Complex multimorbidity**^a^ | 1.33 (0.65-2.75) | 0.438 |
| **Number of Prescription Medicines (Ref: 2-3 medicines)** |  |  |
| 0 medicines | 0.52 (0.18-1.50) | 0.228 |
| 1 medicine | 0.88 (0.40-1.91) | 0.745 |
| 4-5 medicines | 2.00 (1.05-3.84) | 0.036 |
| ≥6 medicines | 1.11 (0.49-2.55) | 0.797 |
| **Urban**^a^ | 1.36 (0.84-2.19) | 0.215 |
| **Partnered**^a^ | 0.78 (0.46-1.31) | 0.344 |
| Intercept | 0.04 (0.00-0.63) | 0.022 |

Note: The variables are mutually adjusted.

^a^Reference groups where not indicated are no LTI scheme eligibility, no private health insurance, male sex, no complex multimorbidity, not urban, and not partnered.

Abbreviations: CI, confidence interval; GMS, General Medical Services; GP, general practitioner; LTI, Long Term Illness

# eTable 8. Univariate logistic model assessing associations with cost-related non-adherence (CRNA)

|  | Reporting CRNA |  |
| --- | --- | --- |
|  | Odds ratio (95%CI) | p value |
| **Any out-of-pocket prescription medicine expenditure (N=5,341)** | 1.45 (0.83-2.54) | 0.192 |
| **Out-of-pocket prescription medicine expenditure (per €100) (N=5,341)** | 1.02 (0.98-1.06) | 0.265 |
| **Any out-of-pocket healthcare (excl. prescription medicines) expenditure (N=5,244)** | 1.13 0.74-1.72) | 0.571 |
| **Out-of-pocket healthcare (excl. prescription medicines) expenditure (per €100) (N=5,244)** | 1.01 (0.99-104) | 0.340 |
| **Healthcare entitlements (Ref: GMS scheme) (N=5,334)** |  |  |
| GP visit card holder | 1.02 (0.51-2.05) | 0.947 |
| Neither GMS scheme nor GP visit card | 0.99 (0.63-1.55) | 0.961 |
| **LTI scheme**^a^ **(N=5,341)** | 1.23 (0.59-2.57) | 0.573 |
| **Private Health Insurance**^a^ **(N=5,340)** | 0.69 (0.46-1.05) | 0.087 |
| **Age (years) (N=5,341)** | 0.99 (0.97-1.02) | 0.574 |
| **Female sex**^a^ **(N=5,341)** | 1.21 (0.79-1.86) | 0.386 |
| **Education (Ref: primary/none) (N=5,341)** |  |  |
| Secondary | 1.33 (0.77-2.31) | 0.302 |
| Third/higher | 1.00 (0.56-1.79) | 0.996 |
| **Equivalised household income (ref: quintile 1, €0-€6,000)** |  |  |
| €6,006-€10,000 | 1.14 (0.60-2.17) | 0.684 |
| €10,029-€15,133 | 1.09 (0.57-2.06) | 0.800 |
| €15,142-€24,000 | 0.66 (0.32-1.37) | 0.270 |
| €24,001-€270,000 | 0.54 (0.24-1.19) | 0.127 |
| **Number of conditions (per condition) (N=5,341)** | 1.21 (1.07-1.36) | 0.002 |
| **Number of conditions (ref:0 conditions) (N=5,341)** |  |  |
| 1 condition | 1.49 (0.61-3.60) | 0.379 |
| 2 conditions | 1.87 (0.79-4.41) | 0.155 |
| 3+ conditions | 2.61 (1.17-5.82) | 0.019 |
| **Complex multimorbidity**^a^ **(N=5,341)** | 1.96 (1.29-2.98) | 0.002 |
| **Number of Prescription Medicines (Ref: 2-3 medicines) (N=5,340)** |  |  |
| 0 medicines | 0.58 (0.27-1.21) | 0.146 |
| 1 medicine | 1.00 (0.52-1.91) | 0.995 |
| 4-5 medicines | 1.78 (1.02-3.12) | 0.044 |
| ≥6 medicines | 1.15 (0.59-2.23) | 0.687 |
| **Urban**^a^ **(N=5,200)** | 1.29 (0.83-2.00) | 0.256 |
| **Partnered**^a^ **(N=5,341)** | 0.67 (0.44-1.03) | 0.418 |

Note: The variables are not mutually adjusted.

^a^Reference groups where not indicated are no LTI scheme eligibility, no private health insurance, male sex, no complex multimorbidity, not urban, and not partnered.

Abbreviations: CI, confidence interval; GMS, General Medical Services; GP, general practitioner; LTI, Long Term Illness
